# LRP1B mutation: a novel independent prognostic factor and a predictive tumor mutation burden in hepatocellular carcinoma

**DOI:** 10.7150/jca.53124

**Published:** 2021-05-13

**Authors:** Fahui Liu, Wanyun Hou, Jiadong Liang, Lilan Zhu, Chunying Luo

**Affiliations:** 1 Department of Pathology, Affiliated Hospital of Youjiang Medical University for Nationalities, Baise 533000, Guangxi, PR China.; 2 Youjiang Medical University for Nationalities, Baise 533000, Guangxi, PR China.; 3 Medical College of Guangxi University, Nanning, 530004, Guangxi, PR China.

**Keywords:** hepatocellular carcinoma, bioinformatics, LRP1B, mutation, TMB, prognosis

## Abstract

Hepatocellular carcinoma (HCC) is one of the most common malignancies globally and the second leading cause of cancer-related death. Low-density lipoprotein (LDL) receptor-related protein 1B (LRP1B) is one of the commonly mutated genes in HCC, but its role in HCC remains unclear. In this study, we analyzed the role of LRP1B mutation in HCC. The bioinformatics results show that LRP1B had a frequency of mutation in HCC patients, and LRP1B mutation was associated with a higher tumor mutation burden (TMB), and survival analysis proved that the prognosis of HCC patients with LRP1B mutation was poor. Univariate and multivariate COX regression analysis indicated that LRP1B mutation was an independent risk factor in evaluating HCC patients' prognosis. Correlation analysis showed that LRP1B mutation status was associated with the infiltration of 2 types of immune cells and higher expression of immune checkpoint gene human endogenous retrovirus-H long terminal repeat-associating protein 2 (HHLA2) in HCC patients. In summary, the results show that LRP1B mutation is associated with the higher TMB and poor prognosis of patients with HCC, and it was an independent risk factor for clinical outcomes of HCC patients. LRP1B gene mutations can serve as predictors in HCC patients with higher TMB and higher expression of HHLA2. The results of this study will be beneficial to future studies on targeted therapy and immunotherapy for HCC.

## Introduction

HCC is the sixth most common cancer worldwide and the second leading cause of cancer-related death 1. After decades of exploration, the treatment of liver cancer has undergone a process from surgery, radiotherapy, chemotherapy to targeted therapy, and then to immunotherapy. In recent years, immune checkpoint inhibitors such as the emergence of drugs opened a new way of treating liver cancer. High expression of PD-L1 or containing a higher proportion of infiltrating tumor immune cell or lymphocyte tumor is defined as an inflamed tumor 2. These inflamed tumors are sensitive to immune checkpoint inhibitors, and immunotherapy works better than for others. However, there are still potentially severe adverse reactions in immune checkpoint inhibitors 3, 4. Identifying new immune checkpoints and find new biomarkers to screen effective immunotherapy patients has become a significant field of investigation for the immunotherapy of cancer. Recent studies explored the correlation between immunotherapy response and TMB 5, 6. The results showed that TMB could be used as a biomarker for immunotherapy, and patients with higher TMB were more likely to get benefits from immunotherapy.

According to reports, hepatocellular carcinoma has the third-highest rate of somatic mutations, showing a promising immunotherapy prospect 7-9. However, the detection of TMB in HCC patients still has great limitations, such as high cost and long test cycle, requiring a large number of tumor cells to be tested. It is important to find new immune checkpoints or simple, effective, or substitutable predictors to evaluate the efficacy of immunotherapy of HCC patients.

LRP1B is a gene that encodes a protein that is a member of the LDL receptor family. LRP1B mutation can be used as a biomarker to predict the TMB and efficacy of immunotherapy for melanoma and non-small cell lung cancer. Similarly, LRP1B mutation is also related to the TMB of lung cancer patients 10. In another study, LRP1B mutation and the incidence of lung adenocarcinoma patients with COPD showed a significantly positive correlation 11. However, the role of LRP1B mutation in HCC and its mechanism have not been investigated. Bioinformatics analysis can effectively find key driving genes through big data and purposeful analysis 12-14, and combined with the verification of clinical samples, can effectively provide useful insights for tumor treatment 15. This study will explore LRP1B mutation and its effects on HCC patients and analyze the gene mutation status and the infiltration of immune cell in the tumor immune microenvironment and the relationship between gene mutation status and immune checkpoint gene expression by bioinformatics analysis.

## Materials and Methods

### Data retrieval and processing

The somatic mutation data of 361 HCC patients were downloaded from TCGA (http://portal.gdc.cancer.gov/projects). Meanwhile, corresponding clinical data of 361 HCC patients were downloaded from TCGA. The primary lesion was hepatocellular carcinoma, and patients with incomplete information were excluded. Finally, HCC patients with complete data (n=350) were included for subsequent analysis. Besides, the somatic mutation data of 399 cases of HCC patients from China were obtained from the international cancer genome consortium (ICGC) (http://dcc.icgc.org/) (up to November 27, 2019). In order to further prove the reliability of the experiment, we collected the paraffin-embedded tissues of 3 HCC patients with LRP1B mutation from the Department of Pathology, Affiliated Hospital of Youjiang Medical University for Nationalities as the experimental group, and the paraffin-embedded tissues of HCC patients without LRP1B mutation as the control group. All patients were treated for the first time. None of the patients received radiotherapy or chemotherapy before surgery. None of the patients received targeted anticancer drugs before surgery. The primary lesion was hepatocellular carcinoma. The pathological type was hepatocellular carcinoma. Hematoxylin-eosin (HE) staining was observed under the microscope and confirmed to be hepatocellular carcinoma in all patients' pathological specimens. This study was reviewed and approved by the Ethics Committee of the affiliated Hospital of Youjiang Medical University for Nationalities (No: YYFY-LL-2021-02), and all patients agreed with written informed consents.

### Bioinformatic analysis

The somatic mutation data downloaded from TCGA were detected by VarScan software and then visualized by the GenVisR package 16. The TSV file containing somatic mutations of Chinese HCC patients downloaded from ICGC was annotated with HG19 as the reference genome, and the GenVisR package was used for visualization. Gene expression data of HCC patients downloaded from TCGA were divided into a mutation group and a wild group according to the mutation status of HCC patients. Gene set enrichment analysis (GSEA) 17 was used to identify signaling pathways involved in HCC patients between the LRP1B mutant group and the LRP1B wild group and demonstrated significant differences (P-value<0.05) in the enrichment of MSigDB Collection(c2.cp.kegg.v7.1.symbols.gmt). CIBERSORT was used to assess the proportion of 22 immune cell subtypes in the immune microenvironment of HCC patients. The number of permutations was set at 1000, and P-value<0.05 was considered an accurate sample for calculating immune cell content. The results of CIBERSORT were used to estimate the correlation between 22 types of immune cell infiltration among HCC patients and the difference in the abundance of immune cell infiltration between the LRP1B mutation group and the wild group in HCC patients.TIMER2.0 (http://timer.cistrome.org/) 18 was used to evaluate the expression differences of 40 common immune checkpoint genes between the LRP1B wild group and the LRP1B mutation group. P-value<0.05 was considered statistically significant.

### Validation test for bioinformatics analysis

To further verify the bioinformatics analysis results of the difference in immune cell infiltration between the LRP1B wild group and the mutant group in HCC patients. Immunohistochemistry (IHC) staining and HE staining were then performed. For HE staining, the collected paraffin-embedded specimens were cut into 4 μm sections. Then pathological sections were produced by dewaxing, hematoxylin staining, differentiation, eosin staining and sealing according to routine protocols. Differences in the infiltration of neutrophils in different groups of HCC patients were observed by using HE staining. IHC detected the infiltration of naïve CD4 T cells with CD4 (MaxVision, Cat No.190927062d, 1:300 dilution) and CD45RA (Zsbio, Beijing, China, Cat No. 20101088, 1:300 dilution). Briefly, the collected paraffin-embedded specimens were cut into 4 μm sections, which were deparaffinized, hydrated, and placed in EDTA buffer (pH=8.0) for antigen repair. Then add the primary antibody (CD4, CD45RA) and incubate overnight at 4 °C. Drop the corresponding secondary antibody, and then drop the freshly prepared DAB chromogenic reagent on the slice to develop the color. After dehydration with gradient ethanol and transparent xylene, the sections were sealed with neutral balsam.

### Interpretation of validation test results

IHC staining results were checked using the double-blind method by the pathologist under the microscope to observe and judge the stained sections. The microscopic morphological characteristics of HE staining of neutrophils are that the cell size is like red blood cells. The nuclei are lobulated, and 2-3 lobulations are more often, and the cytoplasm is slightly stained.CD45RA and CD4 are located in the cell membrane. The tumor cell membrane has brown particles as positive. The tumor stroma is not stained, or some lymphocytes are stained. The expression level of cancer tissue is expressed by calculating the cell staining intensity score and the number of positive cells in the four high-power fields of each film. According to the staining intensity score: no staining, 0 points; light yellow, 1 point; brown, 2 points; tan, 3 points. Percentage score of positive cells: no positive cells, 0 points; <10% positive rate, 1 Points; 10%-30% positive rate, 2 points; 30%-70% positive rate, 3 points; >70% positive rate, 4 points. Thus the expression level of CD45RA, CD4 is divided into 0-12 points.

### Statistical analysis

R software (version 4.0.0) was used for statistical analysis and visualization of results. The survival analysis of the correlation between gene mutation and prognosis of HCC patients was analyzed by the Kaplan-Meier method and plotted using the survival package. The log-rank test was used to evaluate the statistical significance of prognosis among different patients. Univariate and multivariate Cox regression analysis was used for survival analysis of the clinical characteristics of HCC patients, including age, sex, histological grade, pathological stage, TMB, and LRP1B mutation status. The Mann-Whitney U test tested the correlation between the mutated gene and TMB. Wilcoxon test was used to identify the correlation between mutated genes and the expression of immune checkpoint genes.In all comparisons, a two-tailed P-value <0.05 was considered statistically significant.

## Results

### Genes with a high frequency of mutations in hepatocellular carcinoma

**Figure 1:**
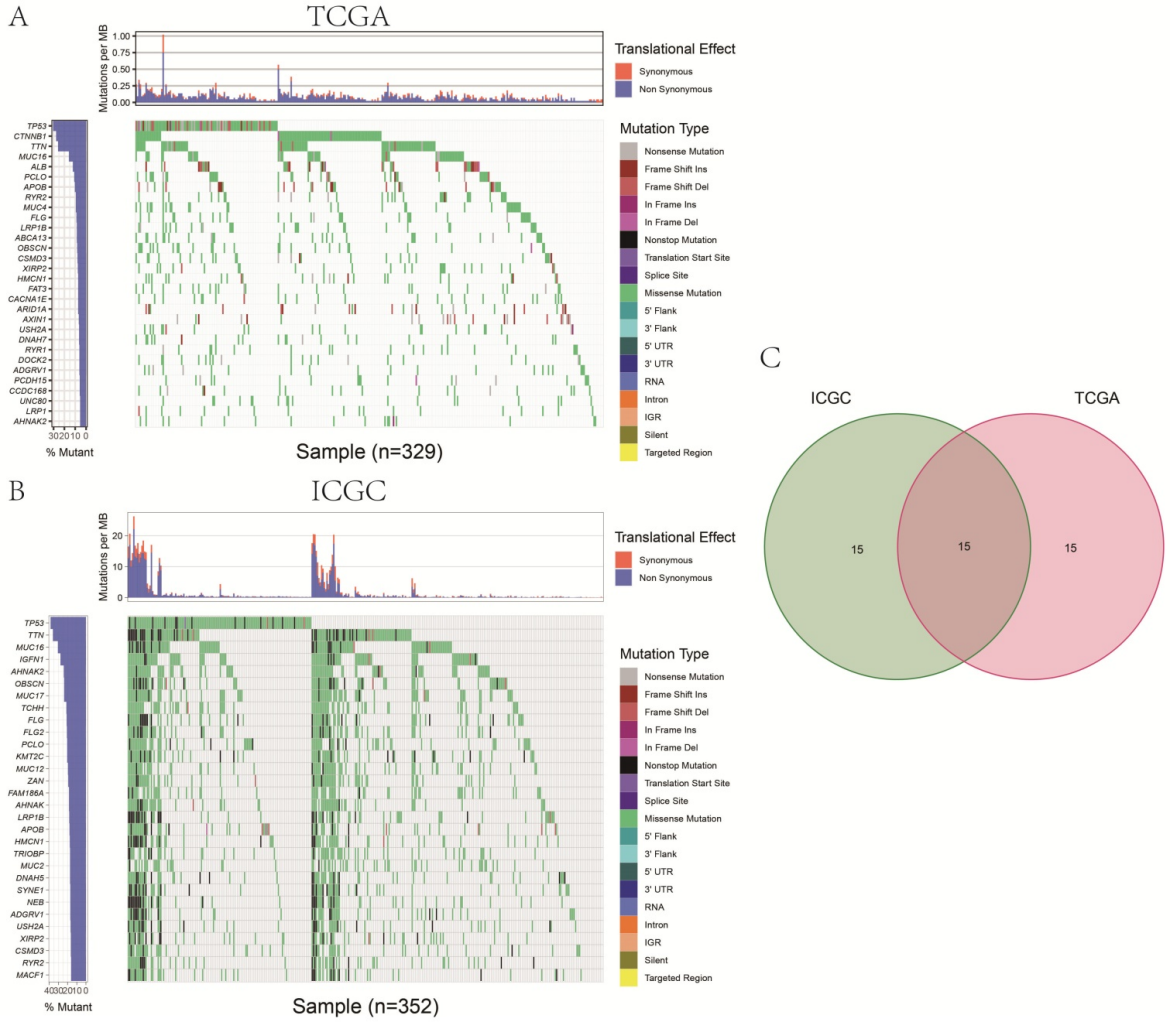
The panel on the left of the two waterfall plots shows the genes with high-frequency mutations in different cohorts, arranged according to their mutation frequency. The panel on the right of the two waterfall plots shows the different types of mutations represented by the various color modules. (A) The 30 genes with the highest mutation frequency in HCC patients in the TCGA cohort. (B) The 30 genes with the highest mutation frequency in HCC patients in the ICGC cohort. (C) The same frequently mutated genes in both TCGA and ICGC cohorts.

We identified 30 genes with the highest frequency of mutation in HCC patients obtained from TCGA (Fig. 1A). In addition, a total of 30 most frequently mutated genes were identified in the database downloaded from the ICGC database (Fig. 1B). Finally, in TCGA and ICGC databases,15 duplicated genes (Fig. 1C), including LRP1B, TP53, TTN, MUC16, AHNAK2, OBSCN, FLG, PCLO, APOB, HMCN1, ADGRV1, USH2A, CSMD3, XIRP2, RYR2, were frequently mutated in HCC patients.

### LRP1B mutation is associated with higher TMB and prognosis in HCC patients

**Figure 2:**
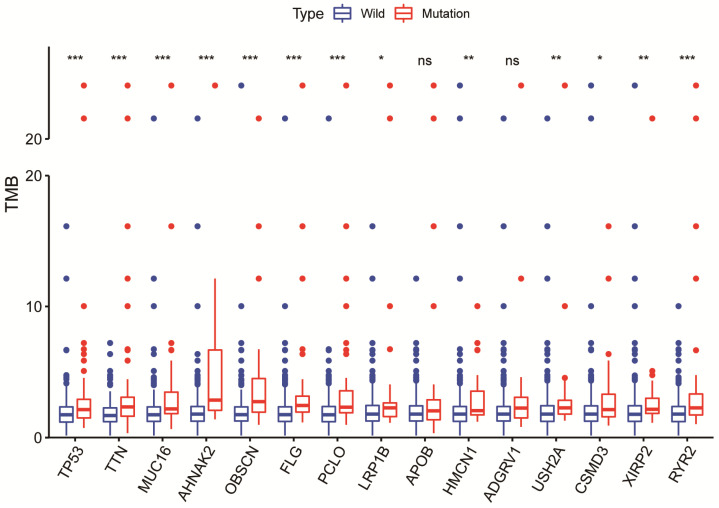
The relationship between gene mutations and a higher TMB in HCC patients. * p<0.05; ** p<0.01; *** p<0.001; ns p>0.05.

**Figure 3:**
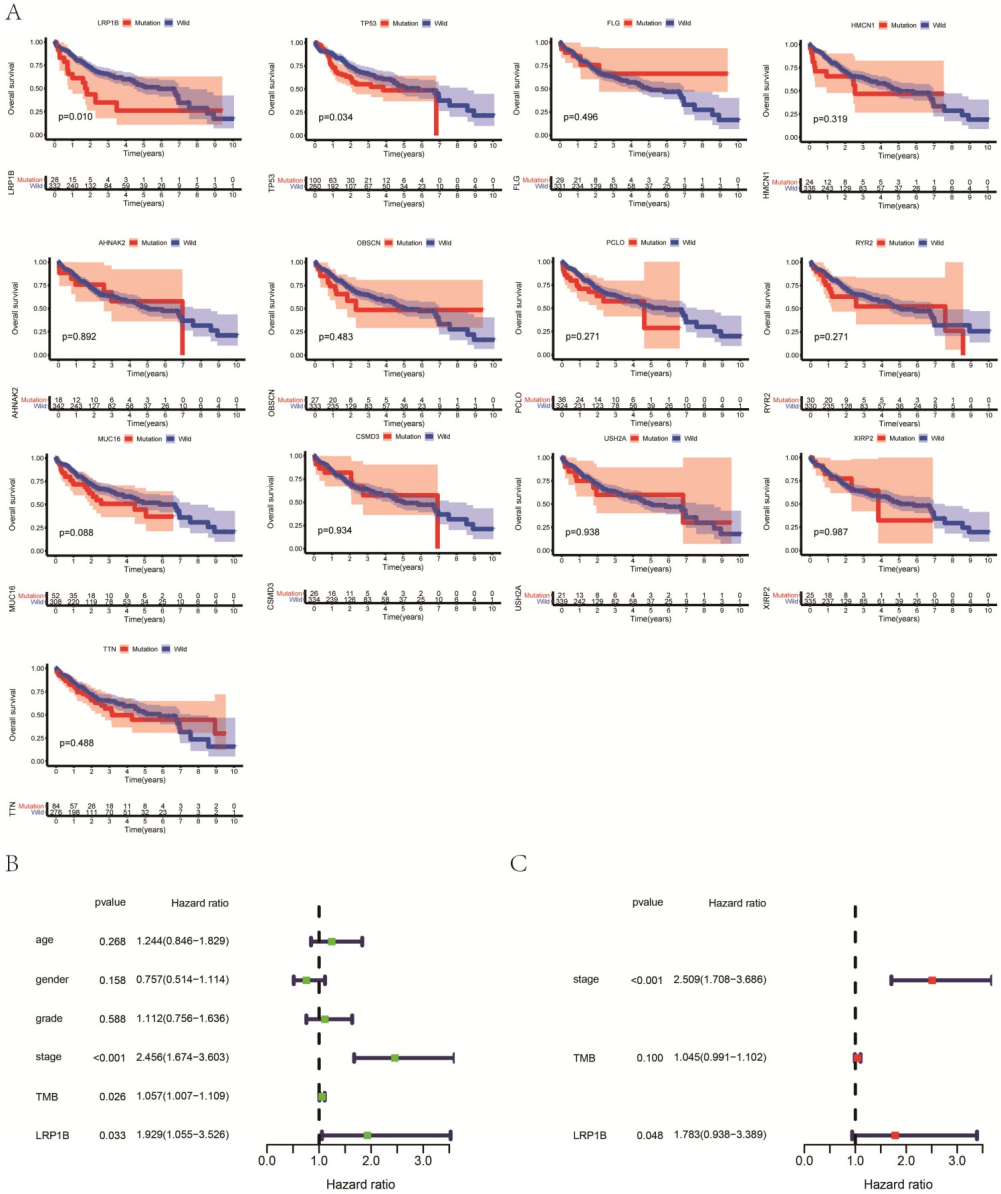
(A) Association of gene mutations with the prognosis of HCC patients. (B) Univariate analyses for HCC patients using the Cox regression model. (C) Multivariate analysis for HCC patients using the Cox regression model.

Among commonly mutated genes, LRP1B, TP53, TTN, MUC16, AHNAK2, OBSCN, FLG, PCLO, HMCN1, USH2A, CSMD3, XIRP2, and RYR2 mutations significantly associated with higher TMB in HCC patients (Fig. 2). Further Kaplan-Meier analysis was performed to explore the relationship between these mutations associated with increased TMB and prognosis in HCC patients. Only the mutation status of TP53 (P=0.034) and LRP1B (P=0.010) is significantly related to the prognosis of HCC patients (Fig. 3A). TP53 mutations exist widely in tumors and have a large number of literature reports, so we choose LRP1B for further analysis. Then univariate regression analysis results identified the pathological stage (P<0.001), LRP1B mutation status (P=0.033), and TMB status (P=0.026) as statistically significant prognostic factors for overall survival in patients with HCC. Multivariable analysis identified both the pathological stage (P<0.001) and LRP1B mutation status (P=0.048) as independent prognostic factors in HCC patients. However, multivariate analysis revealed that TMB status is not an independent risk factor affecting the prognosis of HCC patients (Fig. 3B, 3C).

### Analysis of LRP1B mutation enrichment pathway

**Figure 4:**
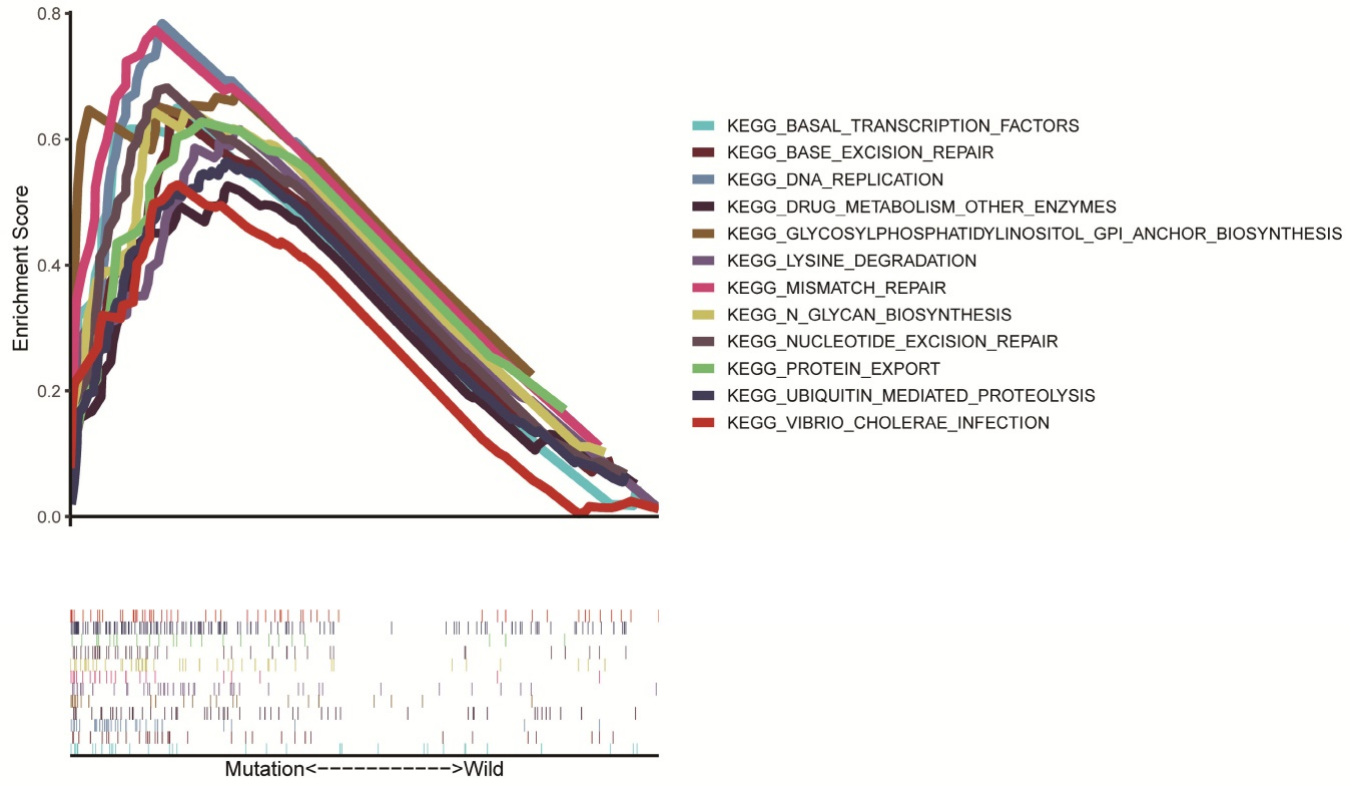
Significant enrichment of KEGG signaling pathways were identified through GSEA in the LRP1B mutation group.

As many pathways are involved in tumor formation, poor survival associated with LRP1B mutation may be associated with many signaling pathways activated in HCC. Through GSEA enrichment analysis, we found that 12 KEGG signaling pathways, including base excision repair, DNA replication, mismatch repair, nucleotide excision repair, basal transcription factors, drug metabolism other enzymes, lysine degradation, protein export, n glycan biosynthesis, glycosylphosphatidylinositol GPI anchor biosynthesis, ubiquitin-mediated proteolysis, vibrio cholera infection showed significantly differential enrichment in LRP1B mutation in the group (Fig. 4).

### The relationship between tumor immune cell infiltration and HHLA2 expression and LRP1B mutation in HCC

**Figure 5:**
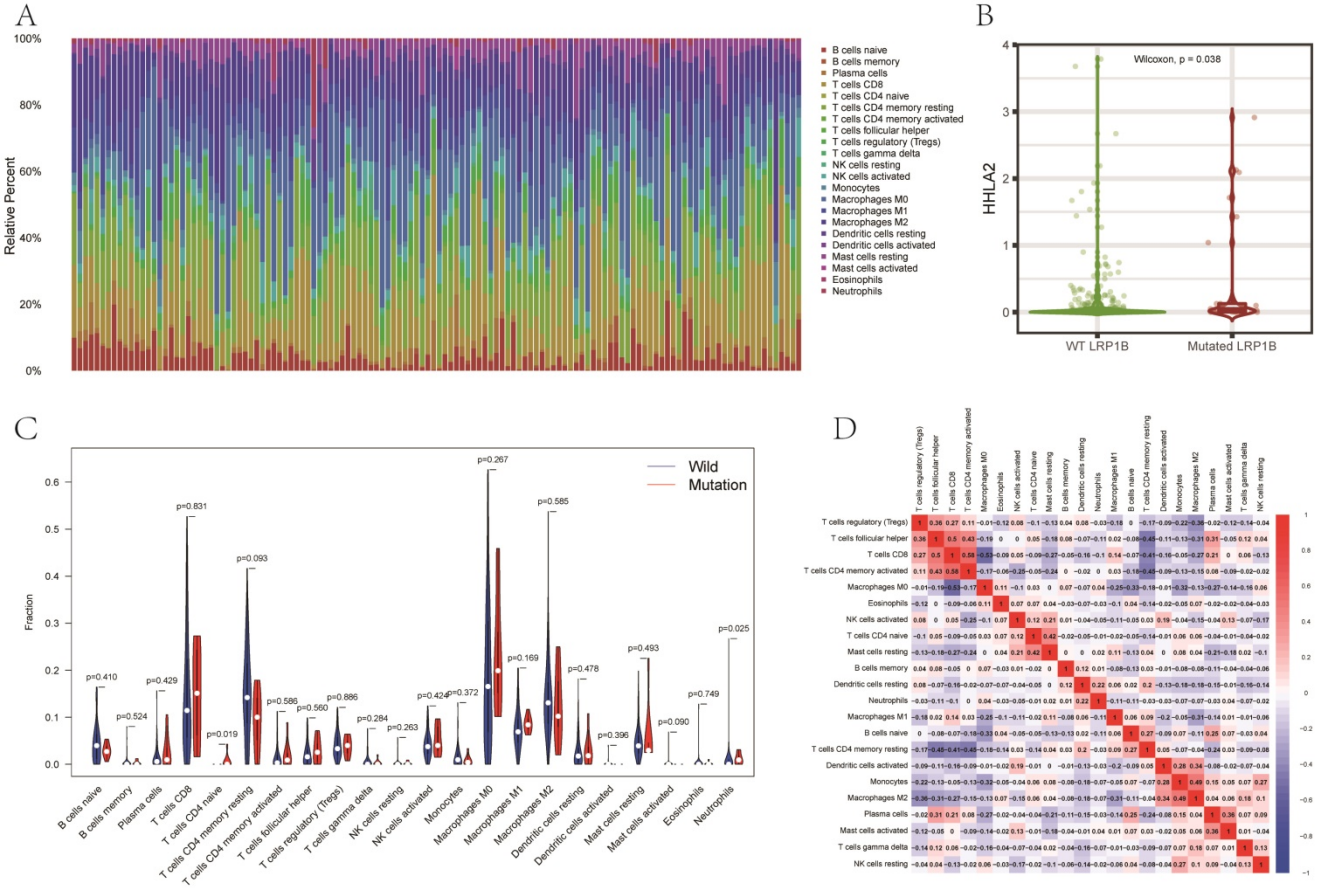
(A) The stacked bar graph shows the distribution of 22 immune cells in each sample. (B) Differences in the expression of immune checkpoint gene HHLA2 between the LRP1B mutant group and the LRP1B wild group. (C) The violin diagram showed differences in the infiltration of immune cells between the LRP1B mutant group and the LRP1B wild group. (D) The correlation matrix of immune cells. Red means positive correlation, and blue means negative correlation.

CIBERSORT was used to analyze the infiltration of 22 kinds of immune cells in the tumor immune microenvironment of HCC patients. The relationship between LRP1B mutation and immune cells was also performed in our research. The results showed significant differences in the composition of 22 kinds of immune cells in each sample (Fig. 5A). The expression level of immune checkpoint genes is an essential biomarker of immunotherapy response. To explore the differences in the expression level of immune checkpoint genes between different LRP1B groups, TIMER2.0 was used to evaluate the differences in the expression levels of several common immune checkpoint genes between the LRP1B mutant group and the wild group. Our results showed that the expression of HHLA2 in the LRP1B mutant group was significantly higher than that in the wild group (P=0.038). (Fig. 5B) Naive CD4 T cells' infiltration was significantly increased in the LRP1B mutant group, while the infiltration of Neutrophil cells in the LRP1B mutant group was significantly lower than that in the wild group (Fig. 5C). However, in other immune cell infiltration analyses, there was no difference in immune cell infiltration between the LRP1B mutant and the wild group. Other analyses showed that naive CD4 T cells had the strongest positive correlation with resting mast cells, and Neutrophils had the strongest positive correlation with resting dendritic cells (Fig. 5D).

### The result of the validation test

**Figure 6:**
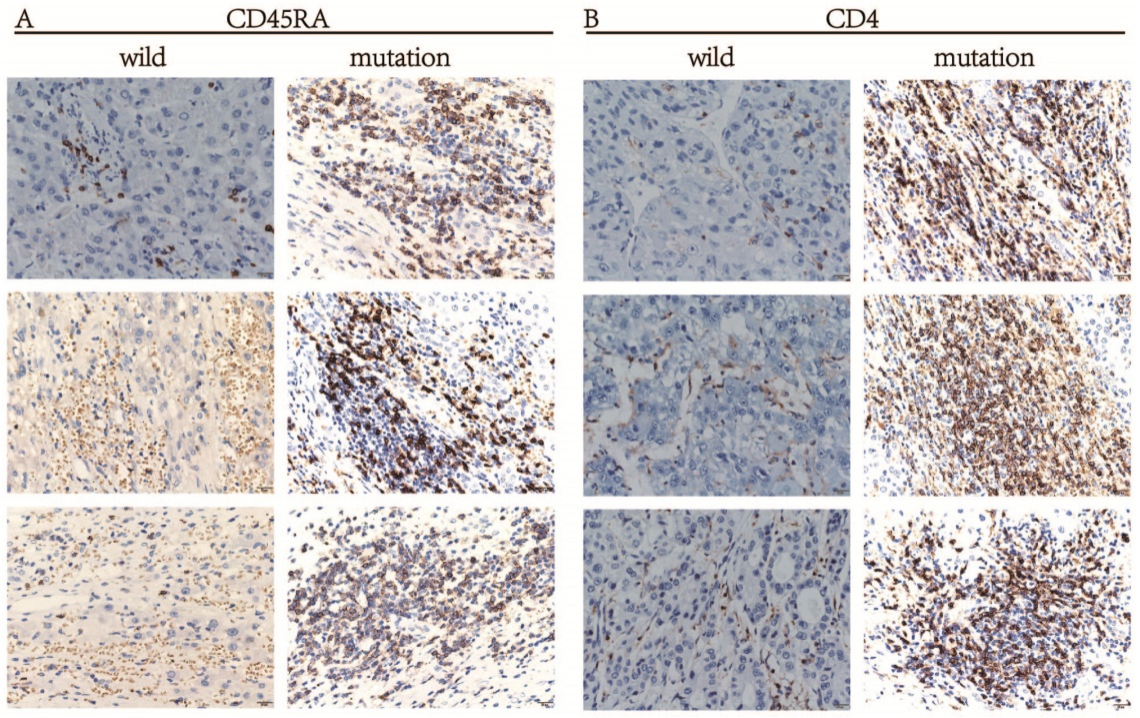
The expression differences of the markers CD45RA and CD4 of Naive CD4 T cells between LRP1B mutant group and wild group in HCC patients. (A) CD45RA; (B) CD4.

**Figure 7:**
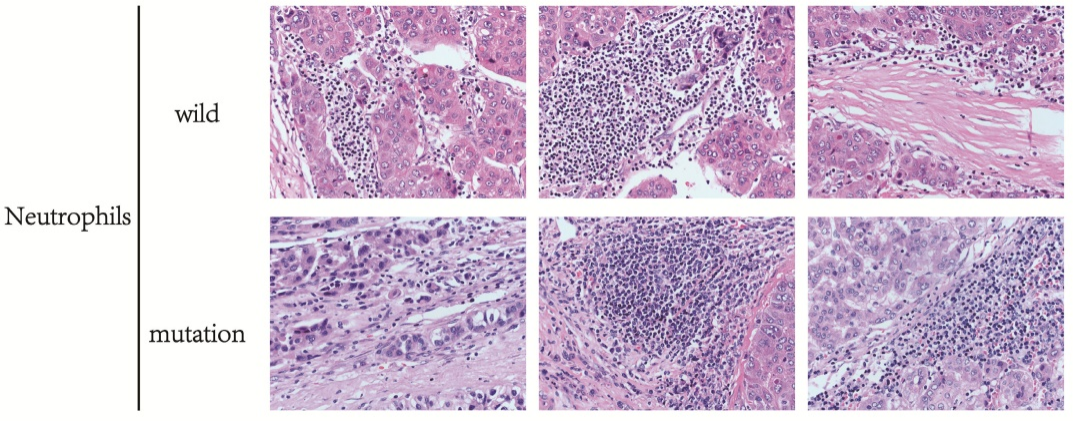
The difference in neutrophil infiltration between HCC patients in LRP1B mutant group and wild group.

A *silico* analysis was performed for the infiltration of immune cells between different groups of patients. To verify the conclusions drawn from the above calculation, HE staining and IHC staining was used to detect the immune cells in the wild and mutant groups of HCC patients. The markers of Naive CD4 T cells in HCC tissues were CD45RA, CD4, and IHC staining results showed that the protein expression level of CD45RA and CD4 was significantly higher in HCC patients with LRP1B mutation than in the wild group (Fig. 6A, 6B), indicating that the infiltration amount of Naive CD4 T cells in the LRP1B mutant group was significantly higher than that in the wild group. In this experiment, we can also see that the infiltration of neutrophils in the LRP1B wild group is higher than in the wild group through HE staining (Fig. 7).

## Discussion

Previous literature has reported the role of LRP1B mutation in some tumors. Missense mutations, nonsense mutations, silencing mutations, frame-shifting deletions, insertions, and frame-missing mutations in LRP1B have been observed in cancers such as lung, skin, and stomach cancer 19. Some studies suggest the critical role of LRP1B as a tumor suppressor gene 20, 21. In our study, we found that LRP1B showed a high mutation frequency in HCC patients. LRP1B mutation is associated with poor prognosis and high TMB in HCC patients. HHLA2 expression in LRP1B mutant group is significantly higher than that in the wild group. These results indicate that LRP1B has a certain research value in HCC.

TMB detection is based on massive next-generation sequencing or whole-exome sequencing. However, some recent studies have reported more straightforward methods, such as mutation detection in a single gene, which can be used to predict TMB in patients 22-24. Therefore, we analyzed the relationship between LRP1B mutation and TMB in HCC patients, and the results showed that LRP1B single-gene mutation was significantly associated with a higher TMB in HCC patients, which proving that LRP1B mutation has a particular effect on predicting TMB of HCC. Similarly, in a recent report, analyzing mutations in a single gene of LRP1B can be used to predict TMB in melanoma patients. TMB of melanoma patients in the LRP1B mutant group was significantly higher than that in the wild group.

Several studies have shown that detecting gene mutations can be used to assess the prognosis of cancer patients. For example, TP53 mutation can be an independent predictor of poor prognosis for NSCLC patients 25, and in lung adenocarcinoma, EGFR mutation is related to poor prognosis in patients 26. Additionally, a recent study has shown that mutations in LRP1B in glioblastoma are associated with patients' poor prognosis 27. Nevertheless, the association between LRP1B mutation and HCC patients' prognosis remains unclear. Based on the above studies, our results for the first time found that LRP1B mutation is correlated with worse prognosis in HCC patients and is an independent risk factor affecting patients' prognosis. To find out the reason lying behind this, we used GSEA enrichment analysis to explore the KEGG pathway promoted by LRP1B mutation, and the results showed that a variety of DNA repair mechanisms, including base excision repair, nucleotide excision repair, and mismatch repair, were promoted in HCC patients with LRP1B mutation. In General, the DNA repair function of normal cells will not be reduced when they are transformed into cancer cells 28. On the contrary, it will significantly increase. The over-activated DNA damage repair system will promote the invasion and metastasis of tumor cells and affect patients' prognosis 29.

Further bioinformatics analysis and HE staining showed that the infiltration of neutrophils in the LRP1B mutant group was significantly lower than that in the wild group. In the tumor microenvironment, neutrophils were divided into two different types of neutrophils, N1 and N2. Neutrophils of N2 type secrete ROS, arginase, peroxidase, and other molecules, inhibit T cells and NK cells' function, and promote the tumor 30. In the process of tumor development, LRP1B mutation may affect the content of N2 neutrophils in HCC patients through some unknown mechanism, which may be one reason for the poor prognosis of patients with LRP1B mutation. Additionally, we also found that the infiltration of naive CD4 T cells in the LRP1B mutation group was significantly higher than that in the wild group by dual means of bioinformatics analysis and pathological verification. Naive CD4 T cells can be divided as Th1, Th2, Th17 cells, etc., after accepting antigen stimulation. Studies have shown that an increase in the proportion of Th17 and Th1 could promote HCC progression and affect patients' prognosis 31. It could also be that the LRP1B mutation group is one of the reasons for the poor prognosis of HCC patients. In summary, we analyzed the potential risk factors affecting HCC patients in the LRP1B mutant group, but the specific situation may require further experiments to confirm.

This study showed a significant increased expression levels of a novel immune checkpoint gene named HHLA2 in the LRP1B mutant group. HHLA2 and PD-L1 are both members of the B7 family with homology and are highly expressed in various solid malignancies 32. As such, HHLA2 is likely to become another crucial immune checkpoint and treatment target following PD-L1. Experiments have proved that HHLA2 has prognostic significance and is more widely expressed as an immune checkpoint compared with PD-L1 in intrahepatic cholangiocarcinoma 33, which prove the feasibility of HHLA2 as a new immune checkpoint in liver tumors. With the development of tumor immunotherapy, more and more immunotherapy predictors were found, such as immune checkpoint gene expression, microsatellite instability, TMB, and DNA mismatch repair defects. Hence, finding more simple and effective immune checkpoint markers has become the key to immunotherapy. Our results show that HCC patients with LRP1B mutations have a higher TMB and a higher expression of immune checkpoint genes, indicating that LRP1B mutations have a certain predictive value in immunotherapy for HCC.

Inevitably, there are still some limitations to our study. First of all, we have not collected the clinical information of HCC patients in the ICGC database, making it impossible for us to verify the correlation between LRP1B mutation and prognosis in different groups. In addition, we did not collect the cohort information of HCC patients currently undergoing immunotherapy, so we could not accurately assess the immunotherapy effect of HCC patients with LRP1B mutation. At the very least, our research results pointed out the important relationship between LRP1B and HCC, and provided further research directions for the prognostic indicators of hepatocellular carcinoma and immunotherapy strategies.

In conclusion, our results showed that LRP1B is characterized by high mutation frequency in HCC patients. Further analysis suggested that LRP1B mutation was significantly related to the poor prognosis of HCC patients. Besides, LRP1B mutation in HCC was significantly associated with a higher TMB and higher expression of immune checkpoint gene HHLA2, providing new evidence to search for more straightforward, complementary, or alternative approaches to TMB as therapeutic markers for immune checkpoint inhibition. Nevertheless, the role and mechanism of the high LRP1B mutation rate in hepatocellular carcinoma are still not fully understood, and we are collecting further data and conducting experimental verification. In conclusion, our study provides new therapeutic targets and research ideas for HCC.

## Abbreviations

HCC: hepatocellular carcinoma
TMB: tumor mutation burden
HHLA2: human endogenous retrovirus-H long terminal repeat-associating protein 2
LRP1B: Low-density lipoprotein receptor-related protein 1B
LDL: Low-density lipoprotein
TCGA: the cancer genome atlas
ICGC: international cancer genome consortium
COPD: chronic obstructive pulmonary disease
PD-L1: programmed death-ligand 1

## References

[1] Bray F, Ferlay J, Soerjomataram I, Siegel RL, Torre LA, Jemal A (2018). Global cancer statistics 2018: GLOBOCAN estimates of incidence and mortality worldwide for 36 cancers in 185 countries. CA Cancer J Clin.

[2] Hegde PS, Chen DS (2020). Top 10 Challenges in Cancer Immunotherapy. Immunity.

[3] Baxi S, Yang A, Gennarelli RL, Khan N, Wang Z, Boyce L (2018). Immune-related adverse events for anti-PD-1 and anti-PD-L1 drugs: systematic review and meta-analysis. BMJ.

[4] Moslehi JJ, Salem JE, Sosman JA, Lebrun-Vignes B, Johnson DB (2018). Increased reporting of fatal immune checkpoint inhibitor-associated myocarditis. Lancet.

[5] Goodman AM, Kato S, Bazhenova L, Patel SP, Frampton GM, Miller V (2017). Tumor Mutational Burden as an Independent Predictor of Response to Immunotherapy in Diverse Cancers. Mol Cancer Ther.

[6] Rizvi NA, Hellmann MD, Snyder A, Kvistborg P, Makarov V, Havel JJ (2015). Cancer immunology. Mutational landscape determines sensitivity to PD-1 blockade in non-small cell lung cancer. Science.

[7] Lu L, Jiang J, Zhan M, Zhang H, Wang QT, Sun SN (2020). Targeting Neoantigens in Hepatocellular Carcinoma for Immunotherapy: A Futile Strategy?. Hepatology.

[8] Obeid JM, Kunk PR, Zaydfudim VM, Bullock TN, Slingluff CL Jr, Rahma OE (2018). Immunotherapy for hepatocellular carcinoma patients: is it ready for prime time?. Cancer Immunol Immunother.

[9] Lu L, Jiang J, Zhan M, Zhang H, Wang QT, Sun SN (2021). Targeting Tumor-Associated Antigens in Hepatocellular Carcinoma for Immunotherapy: Past Pitfalls and Future Strategies. Hepatology.

[10] Lan S, Li H, Liu Y, Ma L, Liu X, Liu Y (2019). Somatic mutation of LRP1B is associated with tumor mutational burden in patients with lung cancer. Lung Cancer.

[11] Xiao D, Li F, Pan H, Liang H, Wu K, He J (2017). Integrative analysis of genomic sequencing data reveals higher prevalence of LRP1B mutations in lung adenocarcinoma patients with COPD. Sci Rep.

[12] Liao Y, Wang Y, Cheng M, Huang C, Fan X (2020). Weighted Gene Coexpression Network Analysis of Features That Control Cancer Stem Cells Reveals Prognostic Biomarkers in Lung Adenocarcinoma. Front Genet.

[13] Liao Y, Xiao H, Cheng M, Fan X (2020). Bioinformatics Analysis Reveals Biomarkers With Cancer Stem Cell Characteristics in Lung Squamous Cell Carcinoma. Front Genet.

[14] Liao Y, Yin G, Wang X, Zhong P, Fan X, Huang C (2019). Identification of candidate genes associated with the pathogenesis of small cell lung cancer via integrated bioinformatics analysis. Oncol Lett.

[15] Zhang W, Gao L, Wang C, Wang S, Sun D, Li X (2020). Combining Bioinformatics and Experiments to Identify and Verify Key Genes with Prognostic Values in Endometrial Carcinoma. J Cancer.

[16] Skidmore ZL, Wagner AH, Lesurf R, Campbell KM, Kunisaki J, Griffith OL (2016). GenVisR: Genomic Visualizations in R. Bioinformatics.

[17] Subramanian A, Tamayo P, Mootha VK, Mukherjee S, Ebert BL, Gillette MA (2005). Gene set enrichment analysis: a knowledge-based approach for interpreting genome-wide expression profiles. Proc Natl Acad Sci U S A.

[18] Li T, Fu J, Zeng Z, Cohen D, Li J, Chen Q (2020). TIMER2.0 for analysis of tumor-infiltrating immune cells. Nucleic Acids Res.

[19] AACR Project GENIE Consortium (2017). AACR Project GENIE: Powering Precision Medicine through an International Consortium. Cancer Discov.

[20] Zhang Z, Cui R, Li H, Li J (2019). miR-500 promotes cell proliferation by directly targeting LRP1B in prostate cancer. Biosci Rep.

[21] Zheng H, Bai L (2019). Hypoxia induced microRNA-301b-3p overexpression promotes proliferation, migration and invasion of prostate cancer cells by targeting LRP1B. Exp Mol Pathol.

[22] Oh JH, Jang SJ, Kim J, Sohn I, Lee JY, Cho EJ (2020). Spontaneous mutations in the single TTN gene represent high tumor mutation burden. NPJ Genom Med.

[23] Zhu G, Pei L, Li Y, Gou X (2020). EP300 mutation is associated with tumor mutation burden and promotes antitumor immunity in bladder cancer patients. Aging.

[24] Wang X, Yu X, Krauthammer M, Hugo W, Duan C, Kanetsky PA (2020). The Association of MUC16 Mutation with Tumor Mutation Burden and Its Prognostic Implications in Cutaneous Melanoma. Cancer Epidemiol Biomarkers Prev.

[25] Jiao X, Qin BD, You P, Cai J, Zang Y (2018). The prognostic value of TP53 and its correlation with EGFR mutation in advanced non-small cell lung cancer, an analysis based on cBioPortal data base. Lung Cancer.

[26] Zhao Y, Pan Y, Cheng C, Zheng D, Zhang Y, Gao Z (2020). EGFR-mutant lung adenocarcinoma harboring co-mutational tumor suppressor genes predicts poor prognosis. J Cancer Res Clin Oncol.

[27] Tabouret E, Labussière M, Alentorn A, Schmitt Y, Marie Y, Sanson M (2015). LRP1B deletion is associated with poor outcome for glioblastoma patients. J Neurol Sci.

[28] Liu J, Renault L, Veaute X, Fabre F, Stahlberg H, Heyer WD (2011). Rad51 paralogues Rad55-Rad57 balance the antirecombinase Srs2 in Rad51 filament formation. Nature.

[29] Chang WH, Lai AG (2019). Transcriptional landscape of DNA repair genes underpins a pan-cancer prognostic signature associated with cell cycle dysregulation and tumor hypoxia. DNA repair.

[30] Giese MA, Hind LE, Huttenlocher A (2019). Neutrophil plasticity in the tumor microenvironment. Blood.

[31] Yan J, Liu XL, Xiao G, Li NL, Deng YN, Han LZ (2014). Prevalence and clinical relevance of T-helper cells, Th17 and Th1, in hepatitis B virus-related hepatocellular carcinoma. PloS one.

[32] Janakiram M, Chinai JM, Fineberg S, Fiser A, Montagna C, Medavarapu R (2015). Expression, Clinical Significance, and Receptor Identification of the Newest B7 Family Member HHLA2 Protein. Clin Cancer Res.

[33] Jing CY, Fu YP, Yi Y, Zhang MX, Zheng SS, Huang JL (2019). HHLA2 in intrahepatic cholangiocarcinoma: an immune checkpoint with prognostic significance and wider expression compared with PD-L1. J Immunother Cancer.

